# Neural correlates of frailty in cognitively healthy adults: A multimodal imaging study

**DOI:** 10.1371/journal.pone.0320492

**Published:** 2025-03-26

**Authors:** Ilaria Parrotta, Lorenza Maistrello, Giorgio Arcara, Dante Mantini, Giovanni Lazzaro, Sonia Montemurro, Nicola Filippini

**Affiliations:** 1 IRCCS San Camillo Hospital, Venice, Italy; 2 Movement Control and Neuroplasticity Research Group, Leuven, Belgium; 3 Department of Philosophy, Sociology, Education and Applied Psychology (FISPPA), University of Padova, Italy; Museo Storico della Fisica e Centro Studi e Ricerche Enrico Fermi, ITALY

## Abstract

**Objective:**

Frailty has emerged as prevalent condition in ageing. While frailty has been assessed through physical and functional criteria, recent studies have explored the link between cognitive decline and frailty, which remains complex and warrants further investigation. Our aims were to compare differences at the brain level between robust and frail older people without dementia and to explore possible associations between brain measures and cognitive performance assessed with neuropsychological tests.

**Methods:**

Using data from the “CAM-Cam” project that recruited a community dwelling population, we identified robust and frail participants based on the Rockwood Frailty index. Magnetic Resonance Imaging was performed to probe the interplay between physical frailty and cognitive health. The main aims were: (i) to identify differences in cognitive performance using the Cattell Culture Fair test and the Tip of the Tongue test and (ii) to assess voxel-wise group-related effects, using a general linear model design to investigate potential differences between our two study groups (“frail” and “robust”).

**Results:**

Our findings revealed significantly smaller grey matter volume in frail individuals, primarily localized in cerebellar areas and in the right supramarginal gyrus. Diffusion magnetic resonance imaging scans showed diminished axial diffusivity values in frail participants, particularly in the corticospinal tract. Resting-state functional MRI showed increased functional connectivity values within the Default Mode Network (DMN) in frail individuals, relative to the robust group in parietal and cerebellar portions of the DMN. Moreover, we observed significant correlations between cognitive score and brain measures for our study groups.

**Conclusions:**

The associations between cognitive test scores and anatomical and functional patterns in the brain highlight the complex interconnections between physical and cognitive aspects of frailty. This study brings novel insights into the early neurobiological markers associated with physical frailty in a cognitively healthy population.

## 1. Introduction

The last decades have seen an increase in the population’s life expectancy. This trend is set to rise over the next 30 years and by 2050 there will be 1.5 billion older adults around the world [1].This demographic phenomenon has also led to the development of age-related and chronic conditions [2]. To counteract this effect, an urgent need of developing preventive medicine technologies, aimed at improving the health status of this population, has surged [3]. Therefore, the identification of those modifiable and non-modifiable factors affecting resilience against and vulnerability to late life pathologies have become a major focus of interest [4].

Ageing is often associated with the presence of frailty. Frailty is a condition characterised by a decrease in homeostatic reserve, which in turn would enhance the vulnerability to both endogenous and exogenous stressor factors like infections, falls or injuries [5,6]. Although frailty is often defined according to physical and functional criteria, several studies have recently evaluated the association between cognitive decline and frailty[7,8]. Scientific evidence suggests a correlation between some aspects of frailty, such as gait speed and handgrip strength with the development of cognitive deficits [9–11]. Pandya and colleagues (2016) have suggested that the onset of cognitive decline does not always occur as a primary manifestation of neurodegenerative disease, which instead may be anticipated by some symptoms more related to a condition of physical frailty [11]. Indeed, in some cases, cognitive functioning remains stable for a long time, probably because it is related to different pathological pathways of ageing, for which specific markers still need to be investigated. Magnetic resonance imaging (MRI) techniques have proved to be sensitive in identifying early markers associated with a graceful or a pathological ageing process [12,13]. Indeed, converging evidence has shown that the ageing process is associated with both structural and functional brain changes, such as brain volume reduction and/or functional reorganization, for the latter with a particular attention on changes occurring within the Default Mode Network (DMN) [14–16]. Indeed, the DMN holds particular significance in ageing and dementia, as its structures are prone to atrophy, amyloid protein deposition, and a general reduction in glucose metabolism and it also appears to play a role in various cognitive processes such as cognitive control, episodic memory retrieval, and imagination [17,18]. Imaging studies have shown significant differences between frail people relative to healthy controls across a variety of MRI-derived measures, both structural and functional [19].

Here, we specifically investigated the role played by physical frailty in a cognitively healthy population. In order to achieve this, we identified a group of robust and a group of frail participants, based on the Rockwood Frailty index [20], among data collected for the “The Cambridge Centre for Ageing and Neuroscience (Cam-CAN)” project [21,22]. Our aims were twofold. Firstly, we investigated whether there was any specific brain difference, either structural or functional, between frail and robust participants in the absence of any sign of cognitive impairment. Secondly, within the frail group, we tested whether both structural and functional brain features were associated with differences in cognitive performance.

## 2. Materials and methods

### 2.1 Study sample

Data used in this study were obtained from the CamCAN repository [21,22]. The participants presented in this study were selected from a larger dataset consisting of 700 individuals evaluated as described in more details in Shafto et al., 2014 and Taylor et al., 2017 [21,22]. Inclusion criteria adopted for this study were the following: 1) age older than 55 years old, 2) mini mental Score Examination (MMSE) score equal to or greater than 25, 3) performance on a specific set of cognitive scores available, 4) Magnetic Resonance Imaging (MRI) Data for structural, diffusion and resting fMRI sequences accessible. frailty was assessed according to the framework outlined by Rockwood at al. [20]. Based on this classification, which refers to the physical efficiency of the participants, we obtained a frailty score which defined the “Frail” and the “Robust” group respectively. more information about frailty index are available in the supplementary Table 1 (Table S1).

**Table1:** Characteristics of the whole sample. MMSE: Mini Mental State Examination, BMI: Body Mass Index, SD: standard deviation, n (%): number and percentage; Cattell Test score refers to the total score obtained to the Cattell test, whereas the Tip of the Tongue score refers to the total correctly retrieved words. ^values are expressed as percentage of total brain volume.

Variable (N = 303)	Frail, N = 78	Robust, N = 225	*p*-value
*Socio-demographic features*			
**Age,** years, mean (±SD)	73 (±9)	70 (±9)	0.017*
**Sex,** female/male, n(%)	43 (55%)/ 35 (45%)	100 (44%)/ 125 (56%)	0.134
**Education**, years, mean (±SD)	19.2 (±3.7)	19.6 (±4.2)	0.612
**Hypertension** no/yes, n(%)	33 (42%)/ 45 (45%)	170 (76%)/ 55 (24%)	<0.001*
**Cholesterol,** no/yes, n(%)	50 (64%)/ 28 (36%)	188 (84%)/ 37 (16%)	<0.001*
**BMI,** mean (±SD)	28.2 (±4.8)	26.4 (±3.9)	0.198
*Cognitive tests*			
**MMSE**, mean (±SD)	28.38 (±1.46)	28.53 (±1.43)	0.419
**Cattell test score**, mean (±SD)	27 (±6)	28 (±6)	0.281
**Tip of the Tongue**, mean (±SD)	12 (±9)	14 (±9)	0.134
*Brain features*			
**Total Brain Volume, cc**	1610.6 (±159.1)	1603.9 (±158.6)	0.748
**Total Gray matter^**	43.99 (±2.04)	44.34 (±2.37)	0.245
**Total White matter^**	35.68 (±1.48)	36.29 (±2.03)	0.015 *

This is a retrospective study of archived data and it received approval for usage for research purposes on the 16th of August 2022. None of the authors had access to information that could identify individual participants during or after data collection. Data collection and sharing for this project was provided by the Cambridge Centre for Ageing and Neuroscience (CamCAN). Individuals unable to give consent were not included.

### 2.2 Cognitive tests

The Mini Mental State Examination (MMSE) score was used to obtain a global index of the cognitive state of each participant. Since the MMSE cannot be considered fully informative in the absence of age-related pathology [23,24], more specific cognitive tasks widely used in ageing research were adopted for assessing cognitive functioning: the Cattell Culture Fair test [25] and the Tip of the Tongue test [26]. These cognitive tests were selected among the other tests present in the original dataset, based both on the interpretability of cognitive functioning assessed and on the tests’ capacity to capture age-related traits associated with cognitive functioning in healthy adults [27]. In the Cattell test participants were asked to perform a nonverbal puzzle involving series completion, classification, matrices, and conditions. It demands participants to carefully select responses from a multitude of choices on each trial and their choices were recorded on an answer sheet. This test (maximum score equal to 46 points) has been widely used for measuring fluid intelligence and it is able to capture high-level cognitive abilities [28]. Prior to performing each subtest, participants received instructions complemented by illustrative examples. The Tip-of-the-Tongue task is especially useful to examine cognitive efficiency in older adults as they may report word finding problems as one of their main concerns [29]. The task includes 50 faces of people from various walks of life (e.g., actors, musicians, politicians, etc), presented in a single pseudorandom order. Participants were instructed to name each person. For each trial, participants viewed a 1000 millisecond fixation cross which was replaced by a picture retained on the screen for 5000 milliseconds. The total of the correctly retrieved names was entered in the analysis models.

### 2.3 MRI Data acquisition and pre-processing

#### 2.3.1 MRI Data acquisition.

MRI scans were performed at the MRC-CBSU on a Siemens 3 Tesla (3T) TIM trio System (Siemens Healthcare GmbH, Erlangen, Germany) equipped with a 32-channel receiver head coil. Briefly, the data used in this study included structural, resting state fMRI (rs-fMRI) and diffusion magnetic resonance images (dMRI) scans. The anatomical scans were acquired using a 3D T1-weighted (T1w) Magnetization Prepared Rapid Gradient Echo (MPRAGE) sequence with the following parameters: Repetition Time (TR) = 2250ms; Echo Time (TE) = 2.99ms; Inversion Time (TI) = 900ms; flip angle = 9°; field of view (FOV) = 256mmx240mmx192mm; voxel size = 1mm isotropic; GRAPPA acceleration factor = 2; acquisition time = 4 minutes and 32 seconds. The rs-fMRI images were acquired using a Gradient-Echo Echo Planar Imaging (EPI) sequence. MRI parameters: TR = 1970ms; TE = 30ms; flip angle = 78°; FOV = 192mmx192mm; voxel-size = 3mmx3 mmx4.44mm. Acquisition time = 8 minutes and 40 seconds, for a total number of 261 volumes acquired. During the rs-fMRI run, participants were instructed to rest with their eyes shut. The dMRI scans were acquired with a twice-refocused spin-echo sequence, with 30 diffusion gradient directions for each of two b-values: 1000 and 2000 s/mm^2^ and three images acquired with a b-value of 0. Other MRI parameters: TR = 9100ms, TE = 104ms, voxel size = 2mm isotropic, FOV = 192mmx192 mm, 66 axial slices, number of averages = 1; acquisition time = 10 min and 2 s. Further details about the MRI protocol can be found in Shafto et al., 2014 and Taylor et al., 2017 [21,22].

#### 2.3.2 MRI data pre-processing.

Data analysis was carried out using FSL tools [30] and Mrtrix3 software [31] for part of the dMRI image pre-processing.

*Anatomical scans:* Pre-processing for structural images included the following steps: (i) reorienting images to the standard (MNI) template, (ii) bias field correction, (iii) brain extraction and (iv) brain tissues segmentation using FMRIB’s Automated Segmentation Tool (FAST). Whole-brain analysis was carried out using a voxel-based morphometry-style analysis (FSLVBM) [32]. Brain extraction and tissue-type segmentation were performed and resulting GM partial volume images were aligned to standard space using first linear (FLIRT) and then nonlinear (FNIRT) registration tools. A study-specific GM template was created. Images were averaged, modulated and smoothed with an isotropic Gaussian kernel of 5 mm Full-Width at Half Max (FWHM) and the GM images were re-registered to the study-specific template, including modulation by the warp field Jacobian.

*Diffusion MRI (dMRI) scans:* pre-processing steps for each subject data consisted of noise-level estimation and denoising (dwidenoise), Gibbs ringing artifacts removal (mrdegibbs), and motion and eddy current-induced distortion correction (dwifslpreproc). Fractional anisotropy (FA), mean diffusivity (MD), axial diffusivity (AD) and radial diffusivity (RD) maps were generated using DTIFit, part of FMRIB’s Diffusion Toolbox, that fits a diffusion tensor model at each voxel [33]. The DTI-derived metrics (i.e. FA, MD, AD and RD) output images were used as input for Tract-Based Spatial Statistics (TBSS), a voxel-wise approach for analysis of DTI metrics data [34]. All subjects’ DTI metrics data were aligned into a common space using FMRIB’s Non-linear Image Registration Tool (FNIRT). The mean FA image was generated and thinned to create a mean FA skeleton, which represents the centres of all tracts common to the group. Each subject’s aligned FA data were then projected onto this skeleton and the resulting data fed into voxel-wise GLM cross-subject statistics.

*Rs-fMRI:* data pre-processing consisted of motion correction, brain extraction, Gaussian kernel smoothing of FWHM of 5 mm, high-pass temporal filtering with a cut-off of 100 s (0.01 Hz), and it was carried out using first-level fMRI Expert Analysis Tool (FEAT) v. 6.00 [35]. FMRI volumes were registered to the individual’s structural scan and standard space images using both FLIRT and FNIRT registration tools, then optimized using boundary-based-registration approach [36]. In order to denoise functional images from the spurious signal and increase the possibility of identifying markers of effective connectivity, FMRIB’s ICA-based X-noiseifier (FIX) was applied [37] and a training dataset specifically developed on the CamCAN dataset. Pre-processed and denoised functional data for each subject were temporally concatenated across all subjects in order to create a single 4D dataset and to derive the population-based resting state-networks (RSNs) using Multivariate Exploratory Linear Optimized Decomposition into Independent Components (MELODIC) [38]. The number of components was fixed to 25 based on an initial analysis of the population using model order estimation, which suggested that only 25 components were significantly non-zero on average [39]. Among the twenty-five derived RSNs (please, see Supplementary S1 Fig for the average group maps), the Default Mode Network (DMN) was selected. This network encompasses the prefrontal cortical areas, anterior and posterior cingulate, lateral parietal and inferior/middle temporal gyri and thalamic nuclei [40]. Pre-processing results of the 303 study participants were visually inspected by a trained neuroscientist (NF) to ensure registration accuracy. The between-subject analysis of the resting data was carried out using the “dual regression” approach, which allows for voxelwise comparisons of resting functional connectivity maps [41].

#### 2.3.3 MRI Statistical analysis – GLM model.

The cross-subject general linear model (GLM) design included the two groups (“frail” and “robust”). Any other socio-demographic variable different between the two study groups, was also added as a nuisance variable (covariate of no interest) to account for potential confounding effects influencing our imaging based results. Voxel-wise GLM was applied using randomise, a permutation-based non-parametric testing (5000 permutations) [42] and Threshold-Free Cluster Enhancement (TFCE) [43], to assess voxel-wise group-related differences. Family wise error (FWE)-corrected cluster significance threshold of *p* <  0.05 was applied to the suprathreshold clusters.

### 2.4 Statistical methods

Summary statistics, namely mean, standard deviation and absolute frequencies (n) and percentages (%), were used to describe the sample characteristics, divided according to the frailty classification. Then, the distribution of variables was investigated by the Shapiro-wilk tests. To assess for the presence of a significant difference in demographic variables, cognitive scores related to frailty and imaging outcomes, we performed tests for comparison for independent data (i.e. Student’s t-test and Mann-Whitney test) or tests for comparison of proportions (i.e. Pearson’s Chi-square test), depending on the nature and the distribution of the variables. Subsequently, Spearman’s test was performed to investigate potential associations between cognitive tests (i.e. Cattell and Tip of the Tongue metrics) and imaging outcomes., All statistical analyses were performed using the R v.4.3.1 software [44], with the significance level set at *p* <  0.05.

## 3 Results

### 3.1 Demographic variables

The mean age of the entire participants was 72.09 years (SD = 8.38) and 47% were females. The age range of the participants was 57–86 years old. Among the total number of participants, 78 (26%) were classified as frail according to the Rockwood criteria. The frail group was significantly older that the robust group. Moreover, as expected and previously reported [45,46], the proportion of participants with hypercholesterolemia and hypertension was significantly greater in the frail group relative to the robust group. No significant group-difference between our study groups was observed for the other socio-demographic features (i.e. sex distribution, years of education and Body Mass Index) or any of the cognitive scores. See Table 1 for a summary of the variables for the frail and robust groups.

### 3.2 MRI results

**Fig 1:**
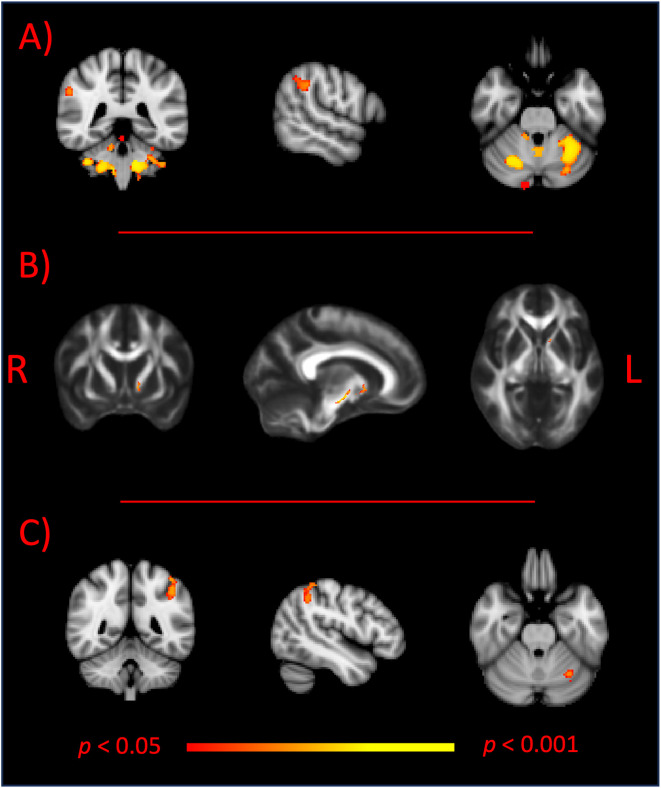
Images depicts group-related differences between frail and robust participants. Red-to-yellow clusters show: (A) reduced Gray Matter density in frail participants relative to robust subjects; (B) reduced axial diffusivity values, derived from diffusion-weighted scans, in frail participants relative to robust subjects; (C) increased functional connectivity within the Default Mode Network (DMN) in frail participants relative to robust subjects. All images displayed here report results with ****p**** <  0.05, corrected for multiple comparisons. R, right hemisphere; L, left hemisphere.

*Anatomical scans:* whole brain analysis revealed significant reduction in grey matter (GM) volume in the “frail” group relative to the “robust” group of participants (Fig 1A). Reporting here and below for each significant difference (i) the cluster size, expressed in number of voxels, (ii) the T-max value for the peak significant area within the reported cluster and (iii) the coordinates of the peak in MNI space, in voxels. Brain regions with group-related differences were mostly localised in cerebellar areas [6434 voxels, T-max: 5.11, MNI coordinates, x–y–z: 56–29–13]. A significant cluster was also found in the right supramarginal (SMN) gyrus [102 voxels, T-max: 5.01, MNI coordinates, x–y–z: 17–44–53]. There were no brain regions in which robust subjects had decreased GM density volume compared with frail participants.

*Diffusion Magnetic Resonance Imaging (dMRI) scans:* whole brain group comparisons showed a reduction in axial diffusivity (AD) values in frail relative to robust participants (Fig 1B). Brain tracts showing group-related differences were found on the lower part of the corticospinal tract bilaterally [338 voxels, T-max 4.80, MNI coordinates, x–y–z: 101-117-61]. There were no brain tracts in which robust subjects had decreased AD values compared with frail participants and there were no significant group differences for any of the other dMRI derived measures (i.e. FA, MD, RD).

*Rs-fMRI scans:* the optimal threshold for the use of FIX, to denoise functional images, was identified as 10 with a mean (median) true positive rate (TPR) and true negative rate (TNR) of 97.2% (100%) and 85.5% (87%), respectively. This is in line or above the suggested thresholding cut-offs (TPR >  95% - TNR >  70%). Significantly increased temporal correlation (coherence) among RSN timeseries within the DMN at rest was observed in frail older adults relative to robust participants in a cluster of regions including the left superior parietal lobule [313 voxels, T-max 4.67, MNI coordinates, x–y–z: 65-42-60] and a smaller cluster located in the cerebellum [67 voxels, T-max 4.46, MNI coordinates, x–y–z: 59-32-23] (Fig. 1C). There were no brain regions in which robust subjects had increased functional connectivity compared with frail participants.

### 3.3 Correlation between MRI measures and cognitive tests

In order to identify whether MRI-related group differences were associated with the performance in the cognitive tests (Cattell and Tip of the Tongue tests), we carried out a partial correlation analysis using the Spearman’s test controlling for the variables age, education, and sex. The results of our analysis revealed significant associations between cognitive variables and MRI results for both the frail and the robust group. For the frail group, positive correlations were observed between the Cattel scores with the cerebellar GM cluster (rho = 0.19; *p* = 0.004) and the SMN GM cluster (rho = 0.16; *p* = 0.018), whereas negative correlations were identified between the Cattel score and the parietal DMN cluster (rho = -0.19; *p* = 0.004) as well as with the cerebellar DMN cluster (rho = -0.21; *p* = 0.001). As for the Tip of the Tongue test, positive correlation was found with the SMN GM cluster (rho = 0.16; *p* = 0.012) whilst negative correlation was observed with the cerebellar DMN cluster (rho = -0.14; *p* = 0.035). With regard to the robust participants, positive correlations were observed between the between the Cattel scores with the cerebellar GM cluster (rho = 0.29; *p* < 0.001) and the SMN GM cluster (rho = 0.23; *p* < 0.001), whereas a negative correlation weas identified between the Cattel score and the cerebellar DMN cluster (rho = -0.08; *p* = 0.040). As for the Tip of the Tongue test, positive correlation was found with the cerebellar GM cluster (rho = 0.32; *p* < 0.001) and the SMN GM cluster (rho = 0.28; *p* < 0.001) whilst negative correlation was observed with the cerebellar DMN cluster (rho = -0.09; *p* = 0.021).

## 4. Discussion

Our results show that, although no differences were found in any of the reported cognitive tasks, the frail individuals presented both morphological and structural brain changes compared to the robust participants. Correlation analysis revealed an association between imaging measures and cognitive performance for both the frail and the robust groups for most of the imaging measures analysed with the exception of the cluster identified in the parietal region of the Default Mode Network (DMN) where significant correlations were observed in the frail but not in the robust group.

In the context of frailty and cognitive efficiency, our results are partly in line with the current literature. Indeed, although some studies reported lower levels of global cognitive efficiency in frail participants compared with robust subjects [47], others did not observe any significant difference [48]. A possible explanation of our results could be related to some socio-demographic characteristics of our study groups. Indeed, the participants assessed in our study were characterized by both a high level of education and Mini Mental State Examination (MMSE) scores. Both these factors may reflect a high level of cognitive reserve which is known to be protective in older age [49,50]. Furthermore participants with high levels of education might have used previously acquired cognitive strategies/resources to counteract brain-related changes and therefore they might have been able to perform adequately on the cognitive tests employed in our study [51]. This may highlight the need of more sensitive screening tests in future studies, in order to stratify a population without dementia through a cognitive screening that includes more executive functions sub-scores, as in the Montreal Cognitive Assessment screening [52,53]. Despite it has been widely reported that physical frailty and cognitive impairment share some pathophysiological mechanisms, such as chronic inflammation and oxidative stress [54], the causal relationship between these two syndromes remains unclear. A review of the literature on frailty and cognitive impairment in neurocognitive disorders, with a focus on executive dysfunction, has shown a strong association between physical frailty and cognitive decline, particularly related to executive functioning in both mild and severe neurocognitive disorders, including Alzheimer’s and Parkinson’s disease [55]. These findings highlight the importance of adopting a multidimensional approach to aging, cognitive decline, and neurodegenerative diseases in order to increase our understanding of the neural mechanisms underlying frailty and its relationship with cognitive decline across the spectrum of neurocognitive disorders [55]. Indeed, it is still uncertain whether specific cerebral markers exist to help us in differentiating cognitive decline associated with unsuccessful aging from that secondary to neurodegenerative diseases [56,57]. More precisely, the increased concentration of pro-inflammatory cytokines can raise the expression of frailty by promoting protein degradation and altering metabolic pathways in the brain [58]. In this context, our results show that brain areas such as the cerebellum and the supramarginal (SMN) area are more prone to reveal a reduction in the volume of grey matter among frail participants, potentially indicating that these regions play a crucial role in the regulation of cognitive and sensorimotor processes. This is in line with previous findings showing that the degree of physical frailty is often proportionally related to the grey matter (GM) thickness [59]. Our structural MRI findings show a reduction in the GM cerebellum and the right SMN gyrus and concurrently a reduction in axial diffusivity (AD) values in frail participants relative to the robust group. The SMN plays a pivotal role in proprioception and it also processes information necessary to define the internal image of our body (body-schema) [60]. Similarly a reduction in AD values may cause impairment in motor control, which could result in an alteration in all the motor criteria of physical frailty. Importantly, a contribution to the expression of the gait impairment could be also secondary to the reduction of GM found in the cerebellum. Indeed, previous findings have shown that a GM volume reduction in the cerebellar area is associated with the onset of all three-motor related features of physical frailty (i.e., grip strength, gait speed and low activity levels), thus playing a pivotal role in one of the cerebral pathways of frailty’s expression and potentially representing an early marker of its onset [61]. Reductions in GM volume in the cerebellum could also be considered an early sign of alteration of executive dysfunctions. Indeed, the interest in cerebellar contribution to social cognition and higher cognitive functions has been rapidly growing over the last years. Two meta-analyses, have shown an activation of distinct cerebellar regions involved in verbal working memory, spatial, executive function, and emotion processing tasks [62,63] and more recently a role in social cognition was also recognized [64]. In other words, it seems that the cerebellum could be able to adapt existing internal models to plan and coordinate actions, which in turn are required for efficient social interaction and interpreting goal-directed actions through the movements of individuals [64]. With regard to resting fMRI data, significant increase of the Default Mode Network (DMN) functional connectivity in the superior parietal lobe and in cerebellar areas was shown for the frail group relative to the robust group. Interestingly, our partial correlation analysis revealed a negative correlation between the Cattel and the Tip of the Tongue scores and the DMN connectivity in the parietal lobe only for the frail group. A possible explanation of this result could be found in the framework of the “functional differentiation”, defined as the age-related phenomenon in which changes occur in the capacity of brain regions to communicate with each other due to a loss of functional specialisation [65,66]. Our result is in line with previous findings indicating an increase in functional connectivity between brain areas, but it is still unclear the exact mechanism through which these alterations could determine a reduced cognitive performance [56,67,68].

Our findings appear to be consistent with neurocognitive dysfunction and frailty patterns observed in the literature, suggesting that neuroimaging may offer important insights into the pathophysiology of frailty and its relationship to cognitive decline in old age. A study on patients with behavioural frontotemporal dementia (bvFTD) examined the relationship between cognitive impairment and frailty status, using both neuropsychological assessments and the Multidimensional Prognostic Index (MPI). These findings showed a significant association between GM atrophy and hypometabolism in areas such as the frontal pole and anterior cingulate cortex, as well as executive dysfunction, mood disturbances, and early frailty. These results suggest that early frailty, particularly characterized by cognitive and executive dysfunction, may heighten the risk of poor outcomes in neurodegenerative diseases [69].

Strengths and limitations for our study should be considered. Our findings adds new insights into the correlation between cognitive performance and changes in brain imaging in an older population with a frail profile. Whereas most of the correlations between cognitive scores and image-derived values were significant for both study groups, which underlies the relationship between brain and cognitive changes regardless of the level of frailty, the parietal portion of the DMN was significantly associated with both the Cattell and the Tip of the Tongue scores only for the frail group, which may reflect a potential role played by this specific brain area in counteracting the frailty status. Indeed, our results strengthen the hypothesis that the functional de-differentiation could be an expression of unsuccessful ageing, since the correlation between cognitive score and functional connectivity in the parietal portion of the DMN was found only in the frail group compared to the robust group. A limitation of the study is that the two groups are slightly unbalanced concerning age, but it is common to find that people displaying frailty are older than robust participants [70]. Here, we accounted for this group-related difference by adding age as a regressor of no interest to our imaging analyses. However, it is not possible to exclude that age may play a role in our results. Another limitation lies in the cognitive results reported here. Indeed, although the cognitive tests employed in our study provide an overall view of high-level efficiency and executive functioning, they could have been not sensitive enough for allowing a finer cognitive differentiation across physically robust and frail individuals. Future studies should use a more widespread range of cognitive tests in order to evaluate other cognitive domains. Indeed, it is reasonable to assume that the use of cognitive tests assessing functions such as attention, working memory, and visuospatial abilities could have highlighted the differences between the groups observed in the imaging findings. Cognitive adaptive tests targeted for evaluating high-level cognitive performance related to reduced cerebellar GM would be suitable for tailoring intervention programmes aimed at preventing cognitive decline in a physically frail population. Furthermore, future studies should further explore the negative correlation we have reported here between cognitive scores and functional connectivity measures. Indeed, whereas our findings may support the dedifferentiation hypothesis, this cannot be generalised to other cognitive domains other than those associated with cognitive tests employed here. Further investigation using cognitive tests across different domains is warranted. Finally, as for the resting fMRI acquisition participants were asked to lie with their eyes closed, it is possible that some of them might have fallen asleep and this could have influenced our reported DMN-related group differences. Further investigations are required to clarify the role played by this potential confounder. Overall, our results provide comprehensive insights into structural and functional brain differences associated with frailty in older adults. The observed changes in GM volume, diffusion measures, and resting-state functional connectivity may contribute to our understanding of the neurobiological underpinnings of frailty and its association with cognitive performance. Further investigations and longitudinal studies are warranted to elucidate the causal relationships and potential implications for interventions targeting age-related cognitive decline and frailty.

## Supporting information

Table S1 The Frailty Index. In this table the 36 items used to construct the Rockwood frailty index are reported. (PDF)

Fig S1 Average group maps of the Resting-State Networks using a data-driven approach. Networks are shown superimposed on the MNI152 standard space template image. Red-to-yellow colours represent z scores > 2.5. R refers to right, L to left hemisphere, S to superior, and I to inferior. (PDF)
