# Supplementary material for: Neural correlates of frailty in cognitively healthy adults: A multimodal imaging study
Source: PLoS One. 2025 Mar 26;20(3):e0320492. doi: 10.1371/journal.pone.0320492 (PMC11940682; doi:10.1371/journal.pone.0320492)
Supplement: Table S1 — (PDF) [file pone.0320492.s001.pdf]

Tab S1. The Frailty Index. In this table the 36 items used to construct the Rockwood frailty index are reported.

**36 ITEMS FRAILTY INDEX**
**1** *High blood pressure (hypertension)*
**2** *High blood cholesterol (hyperlipidaemia)*
**3** *Angina*
**4** *Hearth attack (myocardial infarction)*
**5** *Cardia arrhythmia/palpitations/irregular heartbeat*
**6** *Varicose veins*
**7** *Migraine*
**8** *Stroke*
**9** *Pulmonary embolism*
**10** *Deep vein thrombosis*
**11** *Other vascular disease*
**12** *Diabetes (not during pregnancy)*
**13** *Thyroid disease*
**14** *Peptic ulcer*
**15** *Meningitis or encephalitis*
**16** *Polyps in the large intesistine*
**17** *Gallstones*
**18** *Pancreatitis*
**19** *Appendicitis*
**20** *Liver disease*
**21** *Hay fever/eczema*
**22** *Asthma*
**23** *Bronchitis/emphysema*
**24** *Arthritis*
**25** *Osteoporosis*
**26** *Tuberculosis*
**27** *Depression requiring treatment*
**28** *Other psychiatric illness*
**29** *Benign growths (non cancer)*
**30** *Cancer*
**31** *Intermittent claudication*
**32** *Motor neurone disease*
**33** *Multiple sclerosis*
**34** *Chronic Bronchitis*
**35** *Problems with your speech, memory or vision which got better after a day?*
**36** *Have you ever fractured any bones?*
