# Supplementary material for: Neural correlates of frailty in cognitively healthy adults: A multimodal imaging study
Source: PLoS One. 2025 Mar 26;20(3):e0320492. doi: 10.1371/journal.pone.0320492 (PMC11940682; doi:10.1371/journal.pone.0320492)

## Supplementary Materials

**Fig S1. Average group maps of the Resting-State Networks using a data-driven approach.** Networks are shown superimposed on the MNI152 standard space template image. Red-to-yellow colours represent z scores >2.5. R refers to right, L to left hemisphere, S to superior, and I to inferior.

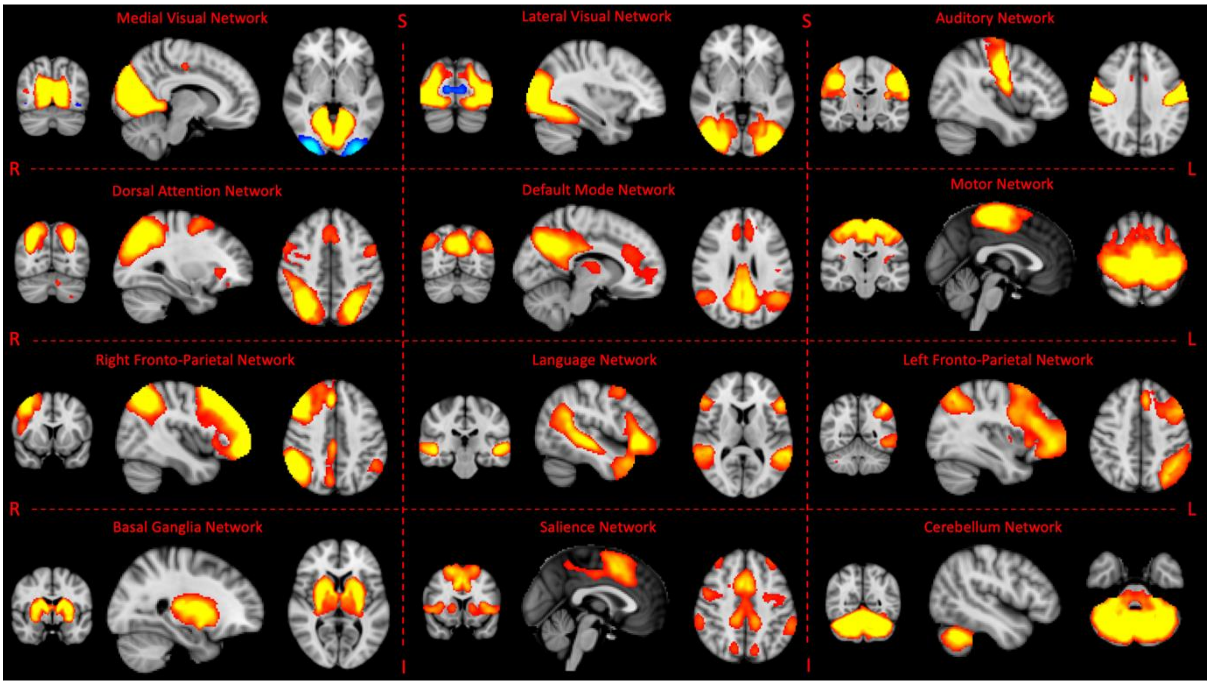

Supplement: Fig S1 — (PDF) [file pone.0320492.s002.pdf]
